# Editorial: Menière's disease: from diagnosis to treatment

**DOI:** 10.3389/fneur.2025.1707265

**Published:** 2025-10-10

**Authors:** Sebastiaan Hammer, Bert De Foer, Athanasia Korda

**Affiliations:** ^1^Department of Radiology, Haga Hospital, The Hague, Netherlands; ^2^Department of Radiology, Algemeen ziekenhuis ZAS Augustinus, Wilrijk, Belgium; ^3^Department of Otorhinolaryngology, Head and Neck Surgery, Inselspital, University Hospital Bern and University of Bern, Bern, Switzerland

**Keywords:** Menière disease, hydropic ear disease, MRI, vestibular test, vestibular migraine (VM)

## 1 Introduction

Menière's disease (MD) is a clinical condition characterized by attacks of vertigo, tinnitus, aural fullness and low-frequency sensorineural hearing loss (1). The etiology of MD remains poorly understood. Although endolymphatic hydrops (EH) is not specific to MD, it is considered a pathological hallmark, as described by Cairns and Hallpike in addition to Yamakawa, who studied temporal bone specimens (2, 3). MD is currently diagnosed using clinical criteria and audiometric testing (4). EH detection, which is now possible using innovative MRI techniques, has proven to be of additional value in selected patients (5, 6). Moreover, vestibular tests may be useful. However, diagnosing MD remains challenging, especially since not all patients present with typical symptoms. Furthermore, there is an overlap with other differential diagnoses, such as vestibular migraine (VM).

This Research Topic aims to address research focusing on developing diagnostic criteria and markers for MD, in addition to treatment strategies, and includes 14 articles. Two studies discussed the methodological needs for studying MD, and defined outcome criteria for MD trials. Ten articles explored the broad spectrum of diagnosing MD using EH and vestibular testing, and differentiated MD from other clinical entities. In addition, two contributions focused on treatment strategies. This editorial synthesizes these studies to highlight the evolving landscape of MD research and care.

## 2 Evolution of symptom patterns and the need for comprehensive management

Pyykkö et al. demonstrated that vertigo attacks generally decline in frequency and severity over time, with spontaneous remission occurring in approximately one-third of patients. They reported on 365 patients, of whom ~34% experienced spontaneous remission of episodic vertigo; 65.5% had persistent balance problems; 34% had vestibular drop attacks (VDAs); 10% had severe falls; and 34.5% had bilateral hearing loss. However, this apparent improvement masks an ongoing burden: VDAs, chronic imbalance, cognitive issues, and emotional distress persist or worsen, particularly in patients with bilateral hearing loss or younger onset. Their work highlights the necessity of a comprehensive approach to management that goes beyond controlling vertigo to encompass fall prevention, hearing support, and psychological care.

## 3 The natural course of intractable MD

In a long-term study, Gerritsen et al. found that even patients with so-called “intractable” MD frequently improved. A total of 35 patients were considered for surgery; of 33 with complete vertigo data 21/33 (64%) were free of vertigo after a median of 5.3 years; 71% eventually declined surgery. These findings emphasize that intractability is often temporary and suggest that overly aggressive ablative treatments should be reconsidered, especially given their irreversible risks.

## 4 Vestibular test dissociation as a diagnostic marker

Mavrodiev et al. provided strong evidence that the dissociation between a reduced caloric response and a normal video head impulse test (vHIT) is a highly specific marker for MD, distinguishing it from vestibular migraine and other vestibular disorders. The authors' total cohort consisted of 2,101 patients (including 627 with definite MD and 473 with VM): caloric–vHIT dissociation had a sensitivity of ≈58.9% and a specificity of ≈83.5%; among patients with MD, the caloric test was abnormal in 71% of cases. Similarly, Cheon et al. worked to define diagnostic thresholds for this dissociation, further standardizing its use in clinical practice. They reported dissociation rates of ~56% in 51 MD patients. Together, these studies establish caloric–vHIT dissociation as a cornerstone diagnostic biomarker.

## 5 Imaging and physiological insights into hydrops

Several groups advanced the understanding of endolymphatic hydrops, which represents the pathological hallmark of MD. Lin H. et al. used wideband acoustic immittance and otoacoustic emissions in animal models to show how hydrops alters cochlear mechanics. Lorente-Piera et al. identified predictors of hydrops in early MD, supporting earlier diagnosis. Venkatasamy and Péporté provided a review of secondary hydrops, underscoring that hydrops can result from trauma, infection, or surgery, and not only from idiopathic MD.

Turning to human diagnostics, Huang et al. proposed optimizing MD staging by integrating electrocochleography with audiometry, while Bernaerts et al. demonstrated the power of hydrops MRI in differentiating MD from vestibular migraine. Together, these advances refine both staging and differential diagnosis.

## 6 Conceptual frameworks and outcome standardization

Chari et al. proposed a modern conceptual framework that integrates pathophysiology, symptom evolution, and patient-centered outcomes into a unified research model. This approach encourages a more personalized understanding of MD trajectories and treatment responses.

In parallel, Boreel et al. led the COSMED study, a scoping review that highlights the variability of outcomes used in MD clinical trials. By advocating for a core outcome set, the authors aimed to standardize trial measures, improving comparability and accelerating treatment advances.

## 7 Surgical controversies: endolymphatic sac surgery

The role of endolymphatic sac surgery remains debated. Gibson reviewed its history, noting reports of short- and long-term vertigo control but at the same time emphasizing the low quality of the evidence and the risks of complications. Given the natural resolution tendencies described by Gerritsen et al. and the variability of outcomes, Gibson's analysis suggests that surgical interventions should be reserved for select cases, ideally within structured clinical trials.

## 8 Clinical variation by age of onset

Lin J. et al. compared early- and late-onset MD, revealing distinctive clinical features that influence disease progression and management needs. Patients with early-onset MD (*n* = 35) and late-onset MD (*n* = 37) reported differences in caloric abnormality rates (20% early vs. 43.2% late) and hydrops severity by age group. Early-onset cases often show more aggressive progression and a greater psychosocial burden, while patients with late-onset MD tend to follow a milder but persistent course. Therefore, the age of onset serves as a valuable stratifier in prognosis and therapeutic planning.

## 9 Differential diagnosis with vestibular migraine

Accurately distinguishing between MD and vestibular migraine remains a central challenge. Beyond the caloric–vHIT dissociation described by Mavrodiev et al. and Cheon et al.. Vosbeek et al. emphasized the value of administering a battery of vestibular function tests to improve diagnostic precision. These insights, together with the hydrops MRI evidence from Bernaerts et al., sharpen clinicians' tools for resolving diagnostic ambiguity.

## 10 Broadening the perspective: beyond vertigo control

While vertigo remains the most dramatic symptom of MD, multiple studies—Pyykkö et al. on symptom evolution, Lin J. et al. on age-related features, and Lorente-Piera et al. on early predictors—emphasized that MD is a multidimensional disease. The persistence of imbalance, cognitive decline, and mental health issues demands a holistic approach to care. Clinicians must support not only symptom suppression but also quality of life in terms of hearing, mobility, and psychological wellbeing.

## 11 Discussion

Collectively, these studies reshape the MD landscape:

- Diagnosis: caloric–vHIT dissociation (Mavrodiev et al., Cheon et al.), hydrops MRI (Bernaerts et al.), and EcochG (Huang et al.) improve accuracy and staging.
- Natural history: longitudinal studies (Pyykkö et al., Gerritsen et al.) show that vertigo often remits, although imbalance and psychosocial symptoms remain.
- Pathophysiology: animal and clinical evidence (Lin H. et al., Lorente-Piera et al., and Venkatasamy and Péporté) deepens our understanding of hydrops.
- Treatment: conceptual frameworks (Chari et al.) and outcome standardization (Boreel et al.) are advancing the quality of clinical trials; however, surgical reviews (Gibson) require caution.
- Phenotypic variation: onset age (Lin J. et al.) and diagnostic differentiation (Vosbeek et al.) refine patient stratification.

## 12 Conclusion

Menière's disease is no longer viewed as a simple vestibular disorder characterized by episodic vertigo. Instead, research led by Pyykkö et al., Gerritsen et al., Mavrodiev et al., Lin H. et al., Cheon et al., Gibson, Chari et al., Huang et al., Bernaerts et al., Lin J. et al., Vosbeek et al., Lorente-Piera et al., Venkatasamy and Péporté, and Boreel et al. paints a picture of MD as a complex, evolving condition requiring multidimensional diagnosis, personalized management, and rigorous research frameworks.
